# Unlocking the brain’s zinc code: implications for cognitive function and disease

**DOI:** 10.3389/frbis.2024.1406868

**Published:** 2024-06-11

**Authors:** Soheila Sabouri, Marzieh Rostamirad, Robert E. Dempski

**Affiliations:** Department of Chemistry and Biochemistry, Worcester Polytechnic Institute, Worcester, MA, United States

**Keywords:** zinc homeostasis, neurodegenerative diseases, zinc transporters, zinc homeostasis in brain, disease

## Abstract

Zn^2+^ transport across neuronal membranes relies on two classes of transition metal transporters: the ZnT (SLC30) and ZIP (SLC39) families. These proteins function to decrease and increase cytosolic Zn^2+^ levels, respectively. Dysfunction of ZnT and ZIP transporters can alter intracellular Zn^2+^ levels resulting in deleterious effects. In neurons, imbalances in Zn^2+^ levels have been implicated as risk factors in conditions such as Alzheimer’s disease and neurodegeneration, highlighting the pivotal role of Zn^2+^ homeostasis in neuropathologies. In addition, Zn^2+^ modulates the function of plasma membrane proteins, including ion channels and receptors. Changes in Zn^2+^ levels, on both sides of the plasma membrane, profoundly impact signaling pathways governing cell development, differentiation, and survival. This review is focused on recent developments of neuronal Zn^2+^ homeostasis, including the impact of Zn^2+^ dyshomeostasis in neurological disorders, therapeutic approaches, and the increasingly recognized role of Zn^2+^ as a neurotransmitter in the brain.

## 1 Introduction

Zinc (Zn^2+^) is one of the most abundant micronutrients in the human body and is essential for life (Maret, 2009). The human body contains between two and 3 g of Zn^2+^. Nearly sixty percent of total Zn^2+^ is found in skeletal muscle, thirty percent in bone, five percent in liver/skin, and the remaining Zn^2+^ is stored in other tissues.

Dietary intake is one of the most important factors that can affect the plasma Zn^2+^ pool (Taylor et al., 1991; Barnett et al., 2013). Zn^2+^ is first absorbed by the small intestine. Organisms dynamically regulate the uptake of Zn^2+^ in the gastrointestinal tract (Takagishi et al., 2017). Considering that Zn^2+^ is water-soluble, Zn^2+^ homeostasis is regulated by endogenous Zn^2+^ secretion rather than by dietary Zn^2+^ absorption (Krebs, 2013). When organisms are Zn^2+^- deficient, the body’s ability to absorb Zn^2+^ increases up to ninety percent. On the other hand, Zn^2+^ is secreted from the gastrointestinal tract or is disposed of through sloughing epithelial cells in the mucosa if Zn^2+^ levels are high (Taylor et al., 1991; Frederickson et al., 2000; Krebs, 2013). If excess Zn^2+^ is taken, for example, with supplements, abdominal cramps and vomiting may occur. These symptoms usually resolve within a few hours. Once absorbed by the gastrointestinal tract, Zn^2+^ is carried through blood by serum albumin which is the most abundant blood plasma protein. Around one percent of organismal Zn^2+^ is found in the blood plasma (Takagishi et al., 2017).

**FIGURE 1:**
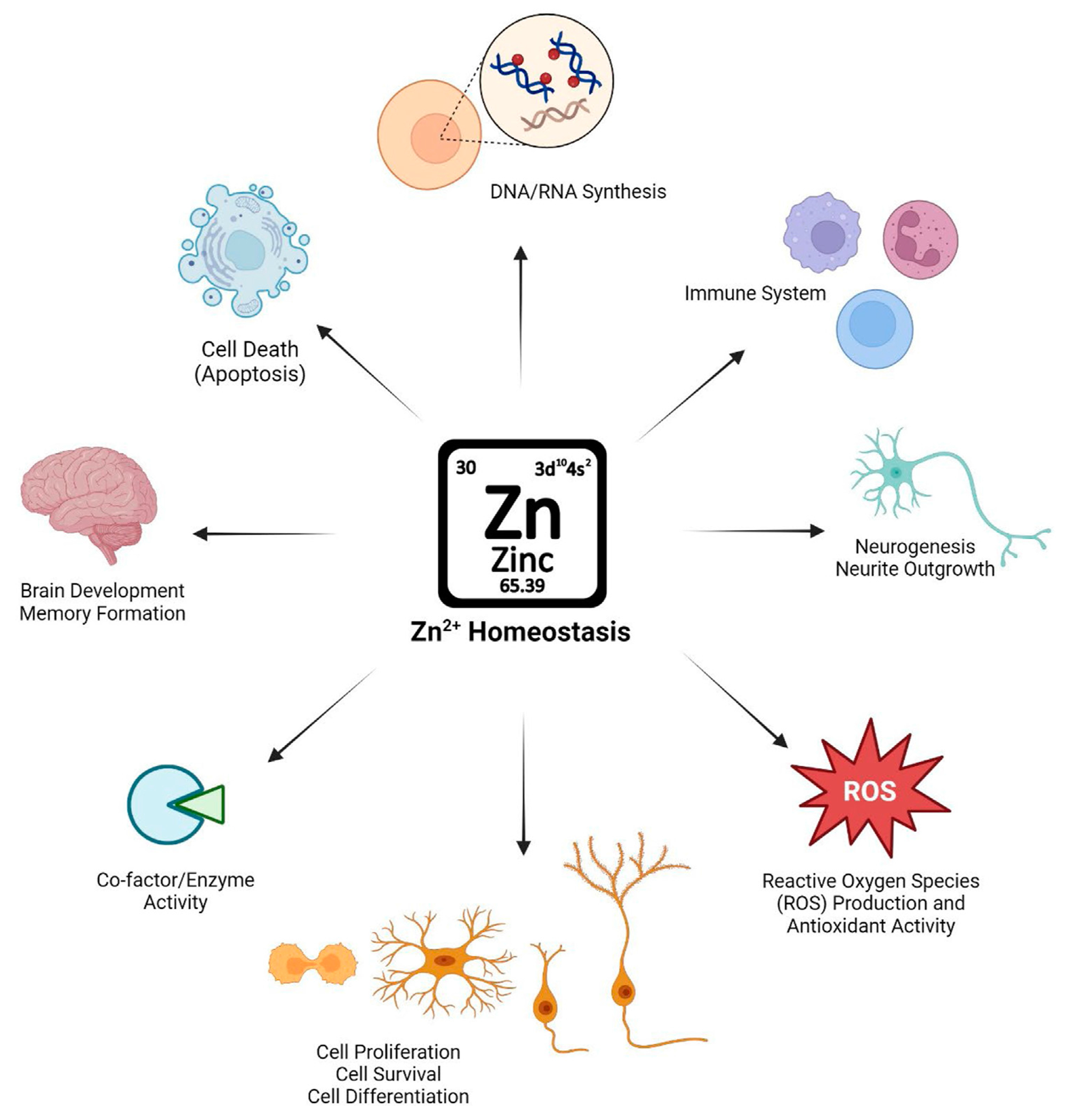
Schematic illustration of the importance of maintaining Zn^2+^ homeostasis in physiological conditions and Zn^2+^’s role in biological systems.

Once dispersed throughout the body, Zn^2+^ has a variety of essential roles, from brain development to apoptosis across different tissues (Figure 1). Zn^2+^ is essential for the structure, stability, and activity of hundreds of human proteins (Maret, 2009). In addition, Zn^2+^ plays an essential role in cellular signaling pathways as well as transcription factors (Hara et al., 2017). In mammalian cells, Zn^2+^ are either bound to proteins or “free.” In the vast majority of mammalian cell types, “free” Zn^2+^ is likely not truly free, but is bound by unknown (non-protein) ligands. The free Zn^2+^ concentration in mammalian cells, while in the picomolar range, likely represents a physiologically significant source of Zn^2+^, particularly regarding its role in signaling.

Neuronal cells are distinct from other cell types as they contain both “free” Zn^2+^ as well as Zn^2+^ which is truly free, i.e., not liganded to biomolecules. This free Zn^2+^ is localized in presynaptic vesicles, such as in the presynaptic vesicles of glutamatergic nerve terminals. These presynaptic vesicles fuse with the plasma membrane upon neuronal activation thereby releasing Zn^2+^ into the synaptic cleft. Release of Zn^2+^ into the synaptic cleft then regulates numerous physiological and pathophysiological functions of the brain, some of which will be described within this review.

## 2 Zn^2+^ homeostatic proteins in the brain

When compared to other organs, there is a high concentration of Zn^2+^ in the brain (~150 μM) (Wang et al., 2020). Zn^2+^ is an essential micronutrient in the central nervous system (CNS) (Takeda, 2001). Differing levels of Zn^2+^ impacts learning, memory, information processing, synaptic plasticity, and regulation of neuronal development (Murakami and Hirano, 2008).

Zn^2+^ is transported through the bloodstream, often bound to carrier proteins like albumin and transferrin to the blood brain barrier (BBB). Once here, plasma membrane transporters facilitate the passage of Zn^2+^ across the blood-brain barrier. Zn^2+^ transport across neuronal membranes, including the BBB, is governed by two families within the solute carrier (SLC) superfamily of proteins. SLCs are one of two major membrane transport proteins superfamilies. SLCs include over 400 member proteins organized into 66 families (Colas et al., 2016). These proteins function to transport a diverse set of substrates including ions and micronutrients across biological membranes. When compared to the second major superfamily of membrane transport proteins [ATP-binding cassette (ABC) proteins], the physiological role of SLCs in human health and disease is not well understood. However, in recent years it has become increasingly recognized that SLCs have important roles in physiological processes. Dysfunction of SLCs can result in both rare and common diseases, thereby opening new opportunities for therapeutic targets (Lin et al., 2015).

Once inside neuronal cells Zn^2+^ is delivered to, or taken from, these transport proteins by a variety of chaperones. These chaperones transfer Zn^2+^ to other proteins including metalloenzymes and metalloproteins. Despite a long-standing search, the identify of these chaperones has long been elusive. However, and as will be described here, recent studies have identified the first intracellular Zn^2+^ chaperone. This provides an exciting opportunity to understand how this essential transition metal moves through cells.

### 2.1 ZnT family in the brain

In humans, SLC30 genes encode ten ZnT transporters (ZnT1-ZnT10) which function to decrease cytosolic Zn^2+^ levels either by transport out of the cell or into intracellular organelles (Hara et al., 2022). ZnTs are part of the larger Cation Diffusion Facilitator (CDF) protein family that contributes to transport of a variety of divalent ions, including Zn^2+^, Mn^2+^, and Fe^2+^ (Montanini et al., 2007). The expression of ZnTs is tightly and dynamically regulated, based on changes in cellular levels of Zn^2+^.

ZnT proteins are expressed dynamically and differentially in different neuronal cell types (see Table 1). ZnT’s in the brain have a critical role in Zn^2+^ homeostatic physiological and pathophysiological prospects. Dysfunction of these proteins have been correlated to numerous diseases. ZnT1 (SLC30A1) is the predominant surface expressed Zn^2+^-exporter in synaptic neurons and glia (Palmiter and Findley, 1995). ZnT1 has been shown to export Zn^2+^ to the extracellular space in the amygdala, hippocampus and parahippocampal gyrus, superior and middle temporal gyrus, inferior parietal lobule, and cerebellum. It is been shown that there is an increase in surface expression of ZnT1 in amygdala, hippocampus/parahippocampal and inferior parietal lobule of Alzheimer Disease (AD) patients (Lovell et al., 2005). In contrast, ZnT1 surface expression is repressed in superior and middle temporal gyrus. Therefore, there is a strong correlation between ZnT1 expression and senile plaques and neurofibrillary tangle levels in amygdala of AD conditions (Lovell et al., 2005). Interestingly, ZnT1 may play a protective role in glia when Zn^2+^ levels are high as pre-treatment with Zn^2+^ induced a four-fold increase in the expression of ZnT1 in astroglia (Nolte et al., 2004). In addition, increasing body mass index (BMI) is correlated with a significant reduction in ZnT1 expression in the brain suggesting a link between ZnT1 and obesity (Olesen et al., 2016).

In contrast to ZnT1, the remaining ZnTs found in the brain are expressed within the membranes of intracellular organelles. ZnT3 is predominantly expressed in the brain and has an outsized role in neurons as ZnT3 transports Zn^2+^ into synaptic vesicles for subsequent release into the synaptic cleft alongside neurotransmitters upon neuronal activation (Ohana et al., 2009; Sensi et al., 2009). Changes in the expression of ZnT3 have been linked to gender-specific susceptibility to AD (Lee et al., 2012). In addition, ZnT3 knockout mice show age-dependent deficits in learning and memory that are evident at 6 months, but not 3 months (Adlard et al., 2010). ZnT4 is expressed in the endolysosomal compartment of neurons. While dysfunction of ZnT4 is best known for resulting in Zn^2+^ deficiency in maternal milk, this protein has also been shown to be expressed in the prefrontal cortex and hippocampus of rats subjected to olfactory bulbectomy, a model of depression (McCormick et al., 2016; Rafalo et al., 2017). ZnT5 is localized to the Golgi apparatus and mediates Zn^2+^ transport that is essential for proper folding of Zn^2+^ binding proteins within this compartment (Suzuki et al., 2005a). ZnT5 is expressed in motor neurons (Pfaender et al., 2016). Zn^2+^ deficiency in motor neurons has been shown to lead to an increase in ZnT5 expression, presumably working towards transporting Zn^2+^ from the early secretory pathway to the cytosol of these neurons thereby counteracting Zn^2+^ deficiency conditions (Pfaender et al., 2016). ZnT6 is expressed in the membrane of the Golgi (Huang et al., 2002).

ZnT10 functions to decrease cytosolic Mn^2+^ levels. Hence, mutations in the ZnT10 gene and/or loss of ZnT10 expression can result in accumulation of Mn^2+^ in cells (Levy et al., 2019). In a novel mechanism, it has been shown that ZnT10 takes advantage of the Ca^2+^ gradient to move Mn^2+^ out of cells. ZnT10 is expressed in the brain and application of external Zn^2+^ downregulates the expression of this transporter (Bosomworth et al., 2012).

### 2.2 ZIP family in the brain

The first Zn^2+^ import transport protein, Zrt1 for Zn^2+^- regulated transporters, was identified in *Saccharomyces cerevisiae* in 1996 (Zhao and Eide, 1996). Shortly thereafter, two genes which encode Fe^2+^ transport proteins, Irt1 and Irt2, for Fe^2+^- regulated transporters, were identified in *Arabidopsis thaliana* (Eide et al., 1996). Following a rapid expansion of sequenced genomes, it was shown that there are hundreds of proteins which are homologous to the Zrt and Irt proteins throughout all kingdoms of life. Together, these proteins comprise the SLC39 group of proteins or ZIPs, for Zrt-, Irt-like Proteins. The ZIP family of proteins have been shown or are hypothesized to increase the cytosolic concentration of transition metals, most often Zn^2+^, but ZIPs can also transport cations including Fe^2+^, Cu^2+^, Ni^2+^, Cd^2+^, and Mn^2+^. These transition metals are transported into the cytosol across the plasma membrane or from intracellular organelles. Within humans, ZIPs have four subfamilies (ZIPI, ZIPII, gufA and LIV-1) and 14 members (ZIP1-ZIP14). LIV-1 is the most common subfamily with a predicted metalloprotease motif (domain V) and HSVFEGLAVGIQ conserved sequence in the fourth transmembrane domain. The conserved sequence is proposed as the main part of Zn^2+^ transport in LIV-1 subfamily members. The second most common subfamily is ZIPII with a conserved sequence (HSVXXGL) in their fourth TMD. Considering that Zn^2+^ transporters tightly regulate cellular Zn^2+^ accumulation, alterations in function of these proteins can result in Zn^2+^ dyshomeostasis and subsequent deleterious outcomes. Between TMDs four and five is a disordered cytoplasmic loop (Bafaro et al., 2015; Bafaro et al., 2019). It is been shown that Zn^2+^ coordination to this domain regulates ZIP surface expression.

ZIP transporters are expressed differentially across neuronal cells (Table 2). While ZIP1 transports Zn^2+^ into post synaptic neurons, ZIP3 transports Zn^2+^ from the synaptic cleft into dentate gyrus (DG) (Bogdanovic et al., 2022). Both ZIP1 and ZIP3 are expressed in hippocampal neurons and deliver approximately half of Zn^2+^ to these cells when Zn^2+^ concentration reaches the low micromolar range (Qian et al., 2011a). Interestingly, deletion of ZIP1 and ZIP3 reduces A1 pyramidal cell injury suggesting that reduced postsynaptic Zn^2+^ entry is a neuroprotectant in these cells.

ZIP4 is the most well understood ZIP transporter. ZIP4 was originally identified as it is expressed in the intestine, the main location of Zn^2+^ uptake. Mutations to this protein can lead to *Acrodermatitis enteropathica*, a Zn^2+^ deficiency disease. However, ZIP4 is also expressed in excitatory synapses where it associates in a complex with postsynaptic scaffold proteins (De Benedictis et al., 2021). Here, it has been suggested that ZIP4 is involved in regulating synaptic Zn^2+^ levels. ZIP6 has been observed both in rat neurons as well as in the human neuroblastoma cell model SH-SY5Y (Chowanadisai et al., 2008).

Neuronal Ceroid Lipofuscinoses (NCL) are fatal childhood neurodegenerative lysosomal diseases. NCL is most often associated with mutations within Ceroid Lipofuscinosis Neuronal (CLN) genes. CLN genes encode thirteen proteins that localize throughout the endomembrane system to regulate a variety of cellular processes. Mutations in CLN genes cause a devastating form of neurodegeneration commonly known as Batten disease (Huber, 2023). It was recently shown that the ER/Golgi-localized ZIP7 colocalizes with CLN6 (Grubman et al., 2014). Under these conditions, the expression level of ZIP7 decreases and it has been hypothesized that loss of ZIP7 may result in subcellular deregulation of biometal homeostasis in NCLs.

ZIP8 and ZIP14 are commonly mentioned together as they both transport Mn^2+^ across biological membranes. ZIP8 is a plasma membrane protein which mediates the uptake of Zn^2+^, Mn^2+^ and Fe^2+^ (Lin et al., 2017). ZIP14 transports Mn^2+^ as well as Zn^2+^into cells (Girijashanker et al., 2008; Fujishiro et al., 2014; Xin et al., 2017). Mn^2+^ is an essential micronutrient in human health as Mn^2+^ imbalances in the brain may cause parkinsonism–dystonia (Roth, 2014). Mutations in ZIP14 have been shown to disrupt Mn^2+^ homeostasis and cause childhood-onset parkinsonism–dystonia (Tuschl et al., 2016). In contrast to what one might expect from deletion of a transition metal importer, when ZIP14 is knocked out in mice, increased Mn^2+^ levels were observed in the brain as was diminished motor activity suggesting that ZIP14 function is an essential factor required to prevent Zn^2+^-linked neurodegeneration (Aydemir et al., 2017).

In contrast to ZIP8 and ZIP14, ZIP12 transports Zn^2+^ transporters and is expressed to a high level in the brain (Takagishi et al., 2017). ZIP12 is essential in the activation of cAMP response element binding protein (CREB) signaling for neuronal differentiation, neurite outgrowth, and tubulin polymerization (Chowanadisai et al., 2013). Although the association of ZIP12 in human diseases is not clear, ZIP12 mRNA is increased in brain regions of schizophrenic patients (Davis et al., 2021). ZIP12 is also thought to have a role in neural tube closure and embryonic development in *Xenopus* tropicalis (Davis et al., 2021).

While many studies in the literature have focused on expression patterns of single ZIP genes, in recent years, more systemic approaches have been utilized to quanitfy changes in expression profiles as undifferentiated cells were differentiated to neurons. For example, mouse fetal Neural Stem Progenitor Cells (NPSC) were cultured with 1.5 μM Zn^2+^ (Mori et al., 2024). Upon differentiation, ZIP1, ZIP4, ZIP12, and ZIP13 were expressed at a higher rate, while ZIP8 was downregulated. In addition, ZnT1, ZnT8 and ZnT10 were upregulated. This provides evidence that the expression profiles of multiple ZIPs are impacted upon neurodifferentiation.

### 2.3 Chaperones

Serum albumin is the major carrier for Zn^2+^ in plasma (Blindauer et al., 2009). Zn^2+^ coordinates to two binding sites within serum albumin, Site A and Site B. Site A of serum albumin has a moderate (micromolar) affinity for Zn^2+^ and therefore this labile pool of Zn^2+^ is responsible for the largest portion of exchangeable plasma Zn^2+^ pool (Kassaar et al., 2015). Recently, it has been suggested that serum albumin delivers Zn^2+^ to ZIPs and that allosteric inhibition of Zn^2+^-binding to albumin by free fatty acids increased Zn^2+^ influx (Coverdale et al., 2022). However, evidence directly linking transfer of Zn^2+^ from serum albumin to ZIPs remains unavailable.

It has been proposed that cellular Zn^2+^ resides in one of three pools: 1) free Zn^2+^ which is uncoordinated in solution, 2) loosely bound Zn^2+^ which can associate and dissociate from biomolecules such as metallothionein, and 3) tightly bound Zn^2+^ which cannot react with other biomolecules or be readily released (Krezel and Maret, 2006). Considering that uncoordinated Zn^2+^ is practically non-existent in most cell types, it has long been recognized that Zn^2+^ is largely bound to proteins and other biomolecules (Vallee, 1959). This requires that once Zn^2+^ is transported across the plasma membrane, there must be chaperones that move Zn^2+^ to its ultimate destination(s). However, identification of these chaperones is better understood in bacterial systems (Kandari et al., 2021).

There have been initial attempts to examine the proteins and mechanism that mediate Zn^2+^ intracellular movement. Zn^2+^ homeostasis in the brain, as in all cells, is tightly controlled by metallothionein’s (MTs). MTs are small, cysteine-rich proteins that play important roles in metal homeostasis and protect cells against heavy metal toxicity, DNA damage and oxidative stress. MTs have the potential to bind multiple transition metals including Zn^2+^ and Cu^2+^. MTs can also coordinate toxic metals such as Cd^2+^ and Hg^2+^. There are 4 MTs expressed in humans (MT1, MT2, MT3 and MT4). Up to seven Zn^2+^ ions can coordinate to MTs in a tetrahedral geometry. MT3 is expressed in astrocytes, cerebellar cortex and Zn^2+^ enriched neurons where it sequesters Zn^2+^ in synaptic vesicles (Masters et al., 1994).

Two groups recently described a family of COG0523 proteins, conserved from yeast to humans, whose members function as Zn^2+^ metallochaperones (Pasquini et al., 2022; Weiss et al., 2022). These proteins, named Zn^2+^-regulated GTPase metalloprotein activator 1 (ZNG1) have been shown to directly transfer Zn^2+^ to type 1 metallopeptidases. This finding provides an exciting starting point to better understand the molecular mechanism by which Zn^2+^ is moved through a cell. In addition, considering that these studies showed that disruption of ZNG1 metallochaperone activity results in decreased cellular proliferation and mitochondrial dysfunction, this is an important starting point to understand to decipher how Zn^2+^ dyshomeostasis can result in disease states.

## 3 Zn^2+^ as a neurotransmitter

Zn^2+^ serves as a crucial signaling molecule within the synaptic cleft, playing multifaceted roles in modulating neurotransmission and synaptic plasticity. Upon neuronal excitation, Zn^2+^ is released from synaptic vesicles alongside neurotransmitters such as glutamate and GABA. Within the synaptic cleft, Zn^2+^ directly activates or modulates a variety of receptors and ion channels, exerting both excitatory and inhibitory effects on synaptic transmission. Through its dynamic regulation of synaptic signaling pathways, Zn^2+^ contributes to the fine-tuning of synaptic activity, synaptic plasticity, and ultimately, neuronal communication within neural circuits. Interestingly, as we will describe below, that while there is recent evidence Zn^2+^ can directly gate a neuronal ion channel the role of this protein in synaptic signaling remains unclear.

### 3.1 Zinc Activated Channels

The Zn^2+^ Activated Channel (ZAC), is encoded by the ZACN gene. ZAC is a Cys-loop receptor (CLR) and comprises its own unique subfamily within the pentameric ligand-gated ion channels (LGIC) as ZAC diverged from neighboring proteins early in evolution. Genes for ZAC are present in humans, zebrafish, and dogs. However, the gene encoding ZAC is a non-functional pseudogene in mouse and rat genomes (Davies et al., 2003; Houtani et al., 2005). ZAC has been shown to be expressed in fetal and adult brain as well as the spinal cord. In addition, tissue-specific expression studies show that ZAC expression coincides with neuronal regions of high Zn^2+^ levels as human (h) ZAC mRNA is present in human hippocampal, striatum, amygdala, and thalamus tissues (Houtani et al., 2005).

The hZAC gene encodes a 411-residue protein with four TransMembrane Domains (TMD) and an extracellular N-terminus domain. This extracellular domain encodes the signature Cys-loop motif. ZAC is a non-selective monovalent cationic ion channel which can be activated by Zn^2+^ and Cu^2+^ (Trattnig et al., 2016). ZAC is the only known human ion channel to be directly activated by transition metals. To date, there is little information on the mechanism or physiological significance of ZAC.

### 3.2 Zinc modulates neurotransmitters

Synaptic vesicles of glutaminergic, glycinergic and GABAergic neurons in the hippocampus possess high concentration of Zn^2+^. In fact, fifteen percent of Zn^2+^ in the brain can be found in synaptic vesicles (Frederickson, 1989). Zn^2+^ is transported into these vesicles by ZnT3. When an action potential reaches the presynaptic terminal, it causes synaptic vesicles to fuse to the plasma membrane of the neuron and release Zn^2+^ as well as co-localized neurotransmitter(s) into the synaptic cleft.

The region most susceptible to Zn^2+^ deficiency in the brain is the hippocampus. Here, Zn^2+^ deficiency results in impaired neuronal proliferation, differentiation, and activation of apoptotic pathways, thus leading to unalterable impairment of learning and memory capacity during early development. In addition, if large quantities of Zn^2+^ are released from the presynaptic cleft to the postsynaptic neurons neurotoxicity can result. Neurotoxicity due to excess Zn^2+^ in the synaptic cleft can also be the result of traumatic injury (Morris and Levenson, 2017).

**FIGURE 2:**
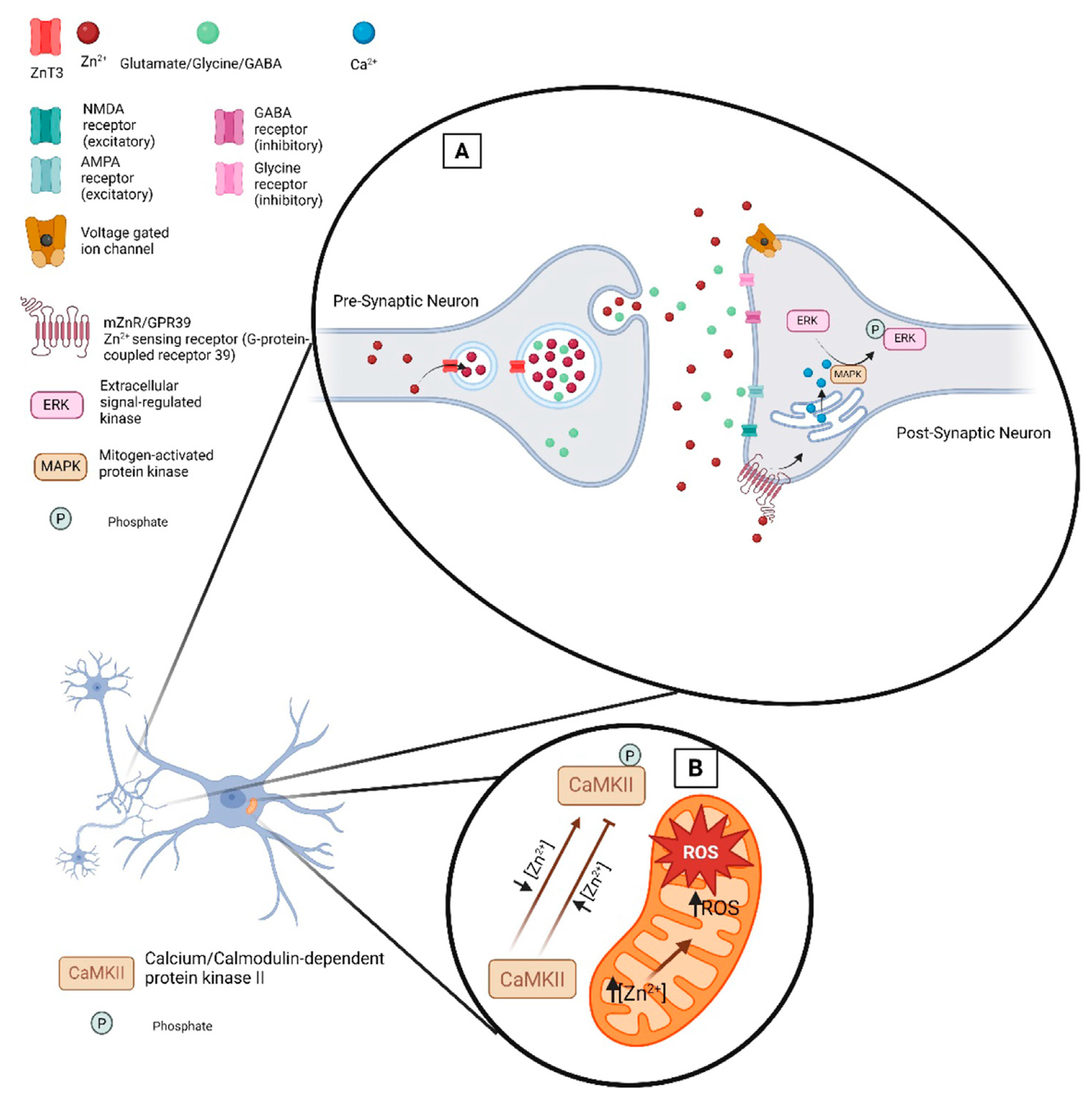
Zn^2+^ at the synaptic cleft. **(A)** Illustration of the proteins that regulate and are impacted by Zn^2+^ at the synaptic cleft. ZnT3 loads Zn^2+^ into presynaptic vesicles where upon neuronal activation it is released into the synaptic cleft alongside neurotransmitters. Zn^2+^ release in the synaptic cleft can modulate excitatory or inhibitory receptors. Transient increases in Zn^2+^ can modulate the function of voltage gated ion channels thereby impacting neuronal excitability. **(B)** Zn^2+^ signaling in ROS level regulation in mitochondria.

In the central nervous system, glutamate is the predominant excitatory neurotransmitter involved in numerous neural functions including learning and memory, long-term potentiation, and synaptic plasticity (Zhou and Danbolt, 2014). In the brain, glutamate binds several receptors. Glutamate receptors are classified into two main subgroups: ionotropic receptors and metabotropic receptors. Ionotropic receptors are transmembrane ligand-gated ion channels while metabotropic receptors act either directly or indirectly as signal transduction enzymes. There are three types of ionotropic glutamate receptors (Figure 2): N-methyl-D-aspartate (NMDA), α-amino-3-hydroxy-5-methy-4-isoxazole propionic acid (AMPA) and kainite receptors. Zn^2+^ directly inhibits NMDA-sensitive glutamate-gated channels by two separate mechanisms: high-affinity binding to N-terminal domains of GluN2A subunits reduces channel open probability, and low-affinity voltage-dependent binding to pore-lining residues blocks the channel (Amico-Ruvio et al., 2011). Synaptically released Zn^2+^ modulates AMPA receptors and impacts fast excitatory neurotransmission and plasticity in glutamatergic synapses (Kalappa et al., 2015). Finally, it is been shown that synaptically released Zn^2+^ inhibits postsynaptic kainate receptors at mossy fiber synapses (Mott et al., 2008). Therefore, disruption of Zn^2+^ transport into synaptic vesicles can have broad impacts on neuronal development.

As a more specific physiological relevant example, it has been shown that Zn^2+^ has multiple roles in how neurons respond to sounds of different volumes (Anderson et al., 2017). First, the addition of Zn^2+^ causes excitatory neurons to increase responses to sounds. Second, addition of Zn^2+^ causes inhibitor neurons to decrease their responses to sounds. Taken together, it was suggested that Zn^2+^ enables the brain to process sounds when moving from one environment to another that has higher or lower sounds.

### 3.3 Zinc as a signaling molecule

Changes in extracellular Zn^2+^ levels impact a myriad of cellular signaling processes. For example, the plasma membrane G protein-coupled receptor 39 (mZnR/GPR39) senses changes in extracellular Zn^2+^ (Hershfinkel, 2018; Xia et al., 2022). Once Zn^2+^ binds to mZnR/GPR39 the ERK/MAPK and PI3K/AKT signaling pathways can be activated. Disruption of this signaling pathway can contribute to neurodegeneration (Abramovitch-Dahan et al., 2016; Khan, 2016; Rychlik and Mlyniec, 2019). Zn^2+^ has been shown to modulate the phosphorylation state of proteins including transcription factors by activating or inhibiting interactions of Zn^2+^ with several enzymes. Therefore, changes in Zn^2+^ levels regulate gene expression and biological outcomes. In addition, Zn^2+^ can function to suppress phosphatase activity and promote phosphorylation reactions. The activation of proteins and subsequent cell signaling pathways are modulated by changes in intracellular levels of Zn^2+^, including proteins such as MAPK, Ca^2+^/calmodulin-activated protein kinase-2 (CaMPK-2), protein kinase C (PKC), P70S6 kinase (P70S6K), cyclic nucleotide phosphodiesterases (PDE), and protein tyrosine phosphatases (PTP) (Figure 2) (Costa et al., 2023).

Changes in cellular Ca^2+^ levels can alter cellular Zn^2+^ levels and *vice versa*. Activation of the ZnR/GPR39 receptor leads to mobilization of Ca^2+^ in the cytosol and ER reservoirs (Sato et al., 2016). Increasing intracellular Ca^2+^ levels can trigger Zn^2+^ release, ROS production and Zn^2+^ waves which are generated from the endoplasmic reticulum (ER). Furthermore, Ca^2+^/calmodulin contributes Zn^2+^ regulation in cells under oxidative stress by NO signal generation. Under oxidative stress conditions, Zn^2+^ initiates antioxidant and repair response to restore cellular balance. Zn^2+^ can also modulate the activation of the major neuronal kinase, serine/threonine-specific kinase, CaMPK-2 (Figure 2) (Poddar et al., 2016). It has been reported that low levels of Zn^2+^ stimulate kinase activity, whereas high concentration of Zn^2+^ inhibits the binding of Ca^2+^/calmodulin and inactivate the substrate phosphorylation activity of CaMPK-2. Moreover, Ca^2+^ and Zn^2+^ shape each other’s intraneuronal dynamics (Dorward et al., 2023).

## 4 Zn^2+^’s role in neurodevelopment

Zn^2+^ plays a pivotal role in neurodevelopment, orchestrating a myriad of processes critical for the formation, maturation, and function of the nervous system. Zn^2+^ is intricately involved in various aspects of neuronal development, including neurogenesis, neuronal migration, synaptogenesis, and myelination. Zn^2+^ also serves as a cofactor for numerous enzymes and transcription factors involved in DNA synthesis, cell proliferation, and differentiation, thereby influencing the generation and organization of neural cells. Dysfunction in Zn^2+^ homeostasis during critical periods of neurodevelopment has been implicated in a range of neurological disorders, underscoring the significance of Zn^2+^ in shaping the structural and functional architecture of the developing brain.

### 4.1 Neurodifferentiation and Zn^2+^

Neurodifferentiation is a multi-stage process that involves morphological and functional changes of precursor cells into mature neurons (Martorana et al., 2018). Neurodifferentiation begins with the proliferation of neural progenitor cells, which subsequently undergo differentiation into various types of neurons and glial cells, each with distinct functions. This complex process involves a series of molecular signals and genetic cues that regulate cell fate determination, migration, and connectivity, ultimately sculpting the intricate circuitry of the brain and spinal cord. Neurodifferentiation plays a critical role in shaping the structure and function of the nervous system, enabling it to carry out a vast array of cognitive, sensory, and motor functions essential for human life. During neurodifferentiation, physical changes are accompanied by the expression of various membrane proteins and receptors (Ernfors et al., 1990).

Transcription factors influence the ability of precursor cells to differentiate into different neuronal cell types and thus impact the formation of different brain areas and sub-structures (Silbereis et al., 2016). Transcription factors are a family of protein molecules that drive gene transcription by binding directly and/or indirectly to upstream genome regulatory elements of protein-coding genes. Among these transcription factors, are Zn^2+^ finger proteins which participate in brain development (Grinberg et al., 2004; Nowick et al., 2009). Zn^2+^ coordinates to Zn^2+^ finger proteins, thereby regulating gene expression. Thus, it should come as no surprise that Zn^2+^ deficiency limits growth in children and can result in mental retardation and learning disabilities. Fortunately, it has been shown that supplemental Zn^2+^ can improve spatial memory, learning and exploratory activities (Piechal et al., 2016).

Among transcription factors, the C_2_H_2_-type Zn^2+^ finger proteins form the largest family in the animal kingdom (Nowick et al., 2010). The C_2_H_2_-type Zn^2+^ finger encodes a consensus sequence, CX_2-4_CX_12_HX_2-8_H where X is any amino acid. These proteins are small peptide domains, which upon Zn^2+^ coordination bind directly or indirectly to upstream DNA sequences to regulate gene transcription. Once Zn^2+^-containing C_2_H_2_-type Zn^2+^ finger proteins are bound to DNA, proteins including cofactors and RNA polymerase II are recruited to initiate and modulate transcription rates of downstream coding sequences (Urrutia, 2003).

Evidence of the importance of C_2_H_2_-type Zn^2+^ finger in neurodifferentiation includes studies showing that Zeb1, a C_2_H_2_-type Zn^2+^ promotes differentiation of radial glial progenitor cells (Yan et al., 2017). Zeb1 performs this function by acting as a transcriptional repressor, thereby regulating proliferation, migration and differentiation. The highest level of expression of Zeb1 are observed during neocortical development and then decreases following birth. Interestingly, genome wide association studies have linked Zeb1 with schizophrenia (Borglum et al., 2014).

ZNF536 is another C_2_H_2_-type Zn^2+^ transcription factor expressed in the brain, including the cerebral cortex, hippocampus, and hypothalamic area (Qin et al., 2009). As P19 cells are differentiated with retinoic acid, it was observed that ZNF536 expression increases. Furthermore, while overexpression of ZNF536 inhibits retinoic acid-induced differentiation, depletion of ZNF539 has the opposite effect. As a consequence of these experiments, it has been proposed that ZNF539 is involved in negative regulation of transcription by RNA polymerase II.

More recent efforts in understanding role of C_2_H_2_-type Zn^2+^ finger transcription factors have included high throughput methods. For example, leveraging the power of CRISPR-Cas in screening all ~1900 human genome transcription factors, identified one C_2_H_2_-type Zn^2+^ transcription factor, ZBTB18, involved in neurodifferentiation (Lu et al., 2023). Loss of this protein resulted in cells which had cytoskeletal defects and stunted neurites/spines.

### 4.2 Neurite outgrowth

Newly generated neurons undergo neurite outgrowth to establish connections with other neurons and form neural circuits. In other words, neurite outgrowth refers to the extension of neurites (axons and dendrites) from neurons (Bostrom et al., 2010). Zn^2+^ is an essential participant in the neurogenesis process (Fidalgo et al., 2011). Neurite outgrowth occurs after neuronal differentiation from stem cell precursors and following the migration of immature neurons from their origin site in the embryo to their final positions. This is an essential step in nervous system development, as it produces new projections for the wiring of neurons.

Zn^2+^ has a direct impact on neurite outgrowth. Using adipose-derived mesenchymal stem cells, which can differentiate into neurons, it was shown that addition of Zn^2+^ promoted outgrowth, while addition of the chelator CaEDTA decreased the level of outgrowth (Moon et al., 2018). On the molecular level, Zn^2+^- enhanced neurite outgrowth was the result of inactivation of RhoA. The activated form of RhoA, V14RhoA, has been shown to inhibit the initiation of neuronal differentiation while the inactivated form of RhoA is necessary for neurite outgrowth (Sebok et al., 1999). Addition of Zn^2+^ promoted the expression of microtubule-associated protein 2 (MAP2) and nestin (NES), two neuronal markers (Moon et al., 2018). Correlated to these results is the observation that mouse neurons produce fewer and shorter neurites after the Zn^2+^ importer, ZIP12, is knocked down (Chowanadisai et al., 2013). Chelation of Zn^2+^ has the same impact as when ZIP12 is knocked down in neurons. In contrast, loading neurons with Zn^2+^ reduced the impact of ZIP12 knockdown neurons on neurite outgrowth.

A C_2_H_2_-type Zn^2+^-finger protein (DISC1-Zn^2+^ finger protein or DBZ) has been implicated in impacting neurite length in PC12 cells. DBZ is expressed solely in the brain of mice and is highly expressed in the cerebral cortex, hippocampus, olfactory tubercle, and striatum (Hattori et al., 2007). In PC12 cells, DBZ co-localizes with disrupted-in schizophrenia (DISC1). These two proteins were shown to interact with each other by immunoprecipitation. Furthermore, expression of DBZ led to a significant decrease of neurite length, implicating DBZ in neurite outgrowth.

## 5 Zn^2+^ as a function of age

Zn^2+^ levels change based on genetics, sex, nutritional intake, health status, physiological conditions, and age (Qu et al., 2020). Serum Zn^2+^ levels in newborn babies (70–150 μg/dL) decrease shortly after birth (60–120 μg/dL) (Hotz et al., 2003). Individuals, less than 10 years of age, with serum Zn^2+^ levels of less than 65 μg/dL are considered to be Zn^2+^ deficient. Above 10 years of age, Zn^2+^ levels are considered to be normal if they are at least 66 and 70 μg/dL for females and males, respectively. Recently, changes in Zn^2+^ levels as a function of aging have been quantified in the Japanese population (Yokokawa et al., 2024). Here it was shown that the proportion of patients with Zn^2+^ deficiency increased with age. Approximately 33% of patients 20–29 years old and 11% of patients in their 80s had a normal range of Zn^2+^ in their blood serum. Among the younger group of patients (20–29 years old), only 16% of them had Zn^2+^ deficiency (<60 μg/dL). However, Zn^2+^ deficiency increased to about 45% among those in their 80s (Yokokawa et al., 2024).

Zn^2+^ levels in the brain change during one’s lifespan (Santhakumar et al., 2018). Zn^2+^ levels remain relatively stable during childhood and adolescence in the brain (Black, 1998). In elderly people, changes in Zn^2+^ metabolism leads to dyshomeostasis of Zn^2+^ in certain brain regions including the olfactory bulb, cerebral cortex, and hippocampus (Sikora et al., 2022). In some regions of the brain (the olfactory bulb), Zn^2+^ levels decrease. While in other parts of the brain, Zn^2+^ levels increase (such as the hippocampus) (Sikora et al., 2022). Excessive Zn^2+^ accumulation in the hippocampus may contribute to synaptic dysfunction and cognitive decline associated with aging. Zn^2+^ dyshomeostasis is related to aging within the cerebral cortex, as well. As the cerebral cortex is involved in high levels of cognitive functions, alterations in Zn^2+^ levels within this area could impact cognitive performance in elderly individuals (Vogler et al., 2022). Fluctuations in Zn^2+^ distribution, transport, and signaling may contribute to age-related cognitive decline, neurodegenerative diseases, and other age-related neuropathological conditions and psychiatric disorders (Takeda et al., 2018; Marchetti et al., 2022). The level of Zn^2+^ in the brain is essential to support neuronal growth, synaptic plasticity, and cognitive development.

## 6 Zn^2+^ and neuronal disease

Considering that Zn^2+^ directly or indirectly impacts such a wide variety of cellular processes within the nervous system, Zn^2+^ dyshomeostasis exerts a profound impact on neuronal diseases including neurotransmission, synaptic plasticity, and oxidative stress responses. Dysregulation of Zn^2+^ homeostasis has been implicated in the pathophysiology of neurodegenerative diseases such as Alzheimer’s disease, Parkinson’s disease, and amyotrophic lateral sclerosis (ALS), where alterations in Zn^2+^ levels contribute to protein misfolding, aggregation, and neurotoxicity. Conversely, Zn^2+^ deficiency or excess has been associated with cognitive impairments, mood disorders, and epilepsy, highlighting the delicate balance required for optimal neuronal function. Understanding the intricate roles of Zn^2+^ in neuronal diseases offers promising avenues for therapeutic interventions aimed at restoring zinc homeostasis and ameliorating neurological dysfunction.

### 6.1 Zn^2+^ and Alzheimer’s disease

**FIGURE 3:**
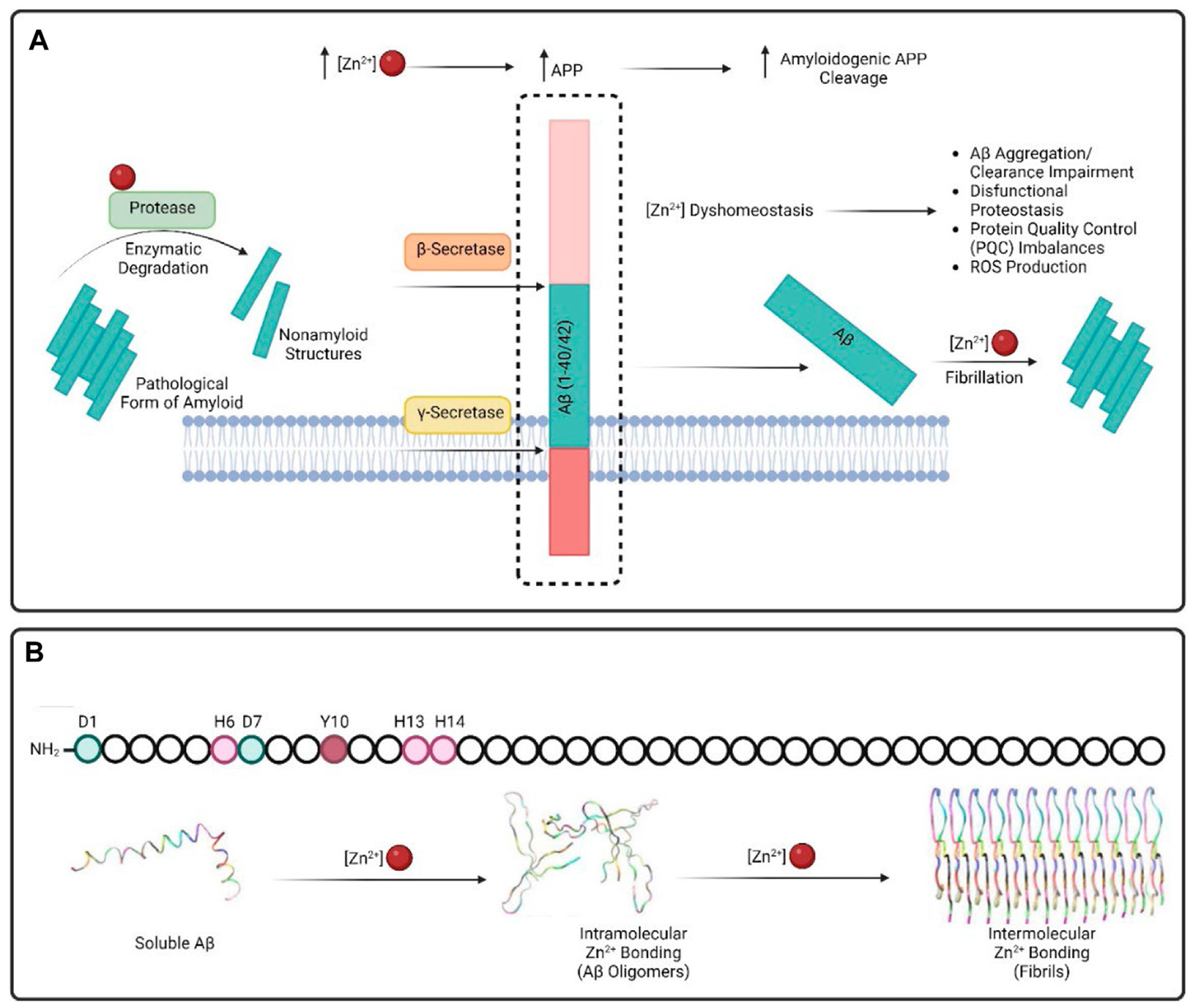
Schematic demonstration of the **(A)** β-amyloid precursor protein (APP) and **(B)** residues which can coordinate Zn^2+^ leading to fibrilization.

The β-amyloid precursor protein (APP) is a single-pass 695 residue transmembrane protein (Figure 3A). APP encodes a large extracellular region and a small intracellular domain. The extracellular domain encodes three distinct domains including E1, KPI and E2. E1 has also been proposed to be important for cell adhesion. The KPI domain is normally expressed in non-neuronal cells (Rohan de Silva et al., 1997). The E2 site can readily dimerize and has multiple metal binding sites (Dahms et al., 2012). The APP can be cleaved by multiple proteases including α-, β-, and γ-secretases. When α-secretase cleaves APP, the product is not amyloidogenic. In contrast when APP is sequentially cleaved by β- and γ-secretases, neurotoxic Aβ peptides are released into the extracellular space. Mutations within APP can result in a decreased rate of cleavage by α-secretase and a subsequent increase in proteolysis by β- γ-secretases. These Aβ peptides can form an oligomeric aggregate, which is the major protein component of amyloid plaques in Alzheimer’s disease (Masters et al., 1985).

Zn^2+^ coordinates with Aβ mainly through N-terminus histidine residues (H6, H13, H14) (Figure 3B) (Minicozzi et al., 2008; Nair et al., 2010; Rezaei-Ghaleh et al., 2011). Zn^2+^ can also coordinate by with Aβ aspartic acid (D1) and tyrosine (Y10) residues (Danielsson et al., 2007; Lee et al., 2018). Aβ self-assembly into an insoluble aggregated form is affected by metal binding to the Aβ peptide. For example, generation of a histidine-Zn^2+^-histidine inter-peptide bridge results in an insoluble aggregate (Miura et al., 2000). In addition, it has been shown that high concentrations of Zn^2+^ enhance Aβ oligomers’ stability thereby increasing cytotoxicity effect of Aβ aggregation. On the other hand, low Zn^2+^ levels may inhibit Aβ oligomerization aggregation.

### 6.2 Cerebral ischemia and hypoxia

When resting, the brain uses around twenty percent of the body’s metabolic energy. This includes about twenty percent of the body’s oxygen supply. Therefore, disrupting the blood supply to the brain can have serious health impacts. Ischemia occurs when blood supply to a specific organ tissue or muscle group is limited. This can lead to a lack of oxygen needed for cellular metabolism. Cerebral ischemia (or brain ischemia) can be a medical emergency that occurs when the brain does not receive enough blood flow to meet metabolic needs.

Cerebral ischemia and hypoxia lead to an abnormally high amount of synaptic Zn^2+^ to be released into the synaptic cleft (Ueba et al., 2018). This can lead to neuronal inflammation and in extreme cases, neuronal cell death. The molecular basis of Zn^2+^- induced neuronal inflammation/death is a complex process and damage can occur through multiple processes. For example, when Zn^2+^ levels are elevated in the synaptic cleft, Zn^2+^ can flow directly into postsynaptic neurons, inducing oxidative stress and resulting in neuronal cell death. In addition, Zn^2+^ can directly damage neurons by activating microglia to produce proinflammatory facts. Finally, increased levels of Zn^2+^ can result in the upregulation of inflammatory proteins which then are toxic to neurons. Interestingly, elevated levels of extracellular Zn^2+^ were reduced in mice with a neuronal-specific ZnT3 knockout (Qi et al., 2023). This suggests that ZnT3 could be a useful target to counteract the impact of elevated Zn^2+^ levels in ischemic neurons.

### 6.3 Kufor-Rakeb Syndrome

Kufor-Rakeb Syndrome is a very rare form of inherited juvenile-onset Parkinson’s Disease (PD). While PD usually affects individuals aged 60 and over, onset of KRS symptoms can be observed prior to age 20. Common symptoms of this disease include bradykinesia (slow movement), rigidity and tremors. Mutations in the ATP12A2 gene result in KRS. ATP13A2 is a P-type ATPase. P-type ATPases use the energy generated from ATP hydrolysis to pump cations and small molecules across biological membranes against their chemoelectrical gradient. ATP13A2 is expressed in intracellular vesicular compartments including lysosomes and early and late endosomes and functions to transport transition metals, including Mn^2+^, Fe^2+^ and Zn^2+^, into lysosomes (Ramirez et al., 2006). Therefore, ATP13A2 functions to prevent transition metal toxicity. Consequently, mutations in ATPA12A2 result in abnormally high cytosolic levels of transition metals leading to toxic effects.

## 7 Zn^2+^ based therapeutic targets

Considering the central role Zn^2+^ plays, either directly or indirectly, in initiating or progressing various disease states, regulating Zn^2+^ levels or the proteins with which Zn^2+^ coordinates in these pathologies may provide a novel avenue towards effective therapeutics. For example, reduced levels of serum Zn^2+^ have been observed in patients with Alzheimer’s disease when compared to healthy controls (Ventriglia et al., 2015). This suggests a linkage between Zn^2+^ dyshomeostasis and AD pathogenesis. This difference could be due to either changes in diet, metabolism or the expression/activities of proteins which regulate Zn^2+^ levels. In fact, there is accumulating evidence that AD coincides with changes in expression levels for proteins that regulate Zn^2+^ influx, efflux and homeostasis. It has been observed that protein levels of ZnT1 were altered in a variety of neuronal cell types in AD patients: Higher in amygdala, hippocampus/parahippocampal gyrus and inferior parietal lobule, while ZnT1 protein levels were lower in the superior and middle temporal gyrus (Lovell et al., 2005). In addition, the expression of ZnT4 and ZnT6 were shown to be higher in the hippocampus/parahippocampal gyrus of individuals with early AD and AD (Smith et al., 2006). ZnT6 is higher in the superior and middle temporal gyrus of AD patients (Smith et al., 2006). The expression of ZIP1 increases as a function of age in the human frontal cortex (Olesen et al., 2016). At the same time, higher mRNA levels of ZIP1 were observed in the cortex of AD patients (Beyer et al., 2012). Similar changes in expression levels of ZIP1 have been seen in a *Drosophila* model of AD leading these authors to suggest that manipulating Zn^2+^ transporters in AD brains could be a novel therapeutic strategy (Lang et al., 2012). Interestingly, MT-3 has been shown to be downregulated in patients with AD (Yu et al., 2001). In addition, MT-3 has been associated with neurodegenerative diseases including amyotrophic lateral sclerosis. Here, overexpression of MT-3 prevented neuronal death and prolonged the life span of mice modeling amyotrophic lateral sclerosis (Hashimoto et al., 2011). MTs have also been implicated in protecting neurons against Parkinson’s disease in mice (Miyazaki et al., 2007).

Considering these molecular changes, clinical trials which have focused on changing levels of Zn^2+^ carry a new level of importance. Considering that excess Zn^2+^ in the synaptic cleft can lead to neuroinflammation and/or death, application of zeolite-based nanomaterials which coordinate Zn^2+^ have been shown to counteract Zn^2+^-induced cerebral ischemia (Huang et al., 2022). Trials which included Zn^2+^ supplements resulted in individuals who had improved performance on cognition tests (Van Rhijn et al., 1990; Potocnik et al., 1997).

## 8 Perspective/conclusion

Zn^2+^ is an essential micronutrient used throughout the lifespan of the human brain. Zn^2+^ is a central participant in neuronal differentiation and as humans age, their levels of Zn^2+^ vary in a cell-type specific manner. These cell type specific changes are largely mediated by changes in ZIP and ZnT expression profiles. However, considering the central role of Zn^2+^-finger transcription factors in regulating cellular homeostasis, these changes in Zn^2+^ levels have an outsized impact on cellular homeostasis. At the same time, there is an increased awareness that changes in Zn^2+^ levels have been shown to be an important microenvironmental risk factor for the development of neurobiological pathologies. Recent studies suggest that there are important interactions between intracellular/extracellular Zn^2+^ levels and neuronal responses that may explain some elements of pathogenesis such as Alzheimer’s Disease. However, the long latency between Zn^2+^ dyshomeostasis and onset of neurological diseases outcome makes it challenging to study these correlations. Enhanced approaches, including new imaging approaches make it plausible to more accurately monitoring Zn^2+^ levels in a time-resolved manner. This provides hope that it may be possible to reduce the incidence of various neurobiological diseases. In addition therapeutics that specifically target Zn^2+^ levels could also lead to new avenues to treat neurological diseases. Thus, it is essential that as investigators learn more about the impact of Zn^2+^ on brain function, one eye is kept towards potential therapeutic approaches.

## Figures and Tables

**TABLE 1:** List of ZnT proteins, including number of amino acids, major locations of expression in the brain as well as sub-cellular location.

Protein	Amino acids	Cellular protein localization	Subcellular location
ZnT1	507 AA	Dendritic spines Xu et al. (2019)	Plasma membrane Nishito and Kambe (2019), Stocks et al. (2021)
		Astroglia, microglia, and oligodendrocytes Nolte et al. (2004)	Basolateral cell membrane Nishito and Kambe (2019)
		Cerebral cortex and cerebellum Sekler et al. (2002)	Cytoplasmic vesicle membrane Stocks et al. (2021)
		Spinal cord Kaneko et al. (2015)	
ZnT2	372 AA	Not known	Not applicable
ZnT3	388 AA	Hippocampus, cerebral cortex, and spinal ganglion Shen et al. (2007)	Cytoplasmic vesicle, secretory vesicle, synaptic vesicle membrane Hildebrand et al. (2015)
		Astroglial cells, and amygdala Lee et al. (2011)	Late endosome membrane Falcon-Perez and Dell Angelica (2007)
			Lysosome membrane Falcon-Perez and Dell Angelica (2007)
ZnT4	429 AA	Spinal cord Kaneko et al. (2015)	Late endosome membrane Falcon-Perez and Dell Angelica (2007)
		Cerebellum Wang et al. (2005)	Lysosome membrane Falcon-Perez and Dell Angelica (2007)
		Cerebral cortex Hasna et al. (2019)	
ZnT5	765 AA	Spinal cord Kaneko et al. (2015)	Golgi apparatus, Golgi stack membrane Kambe et al. (2002), Hoch et al. (2012)
			Cytoplasmic vesicle, secretory vesicle membrane Kambe et al. (2002)
			Cytoplasmic vesicle, COPII-coated vesicle membrane Suzuki et al. (2005b)
ZnT6	461 AA	Spinal cord Kaneko et al. (2015)	Golgi apparatus, trans-Golgi network Ohana et al. (2009)
		Cerebellum Wang et al. (2005)	
ZnT7	376 AA	Spinal cord Chi et al. (2008), Kaneko et al. (2015)	Golgi apparatus membrane, cytoplasmic vesicle, sarcoplasmic reticulum, mitochondrion Suzuki et al. (2005a)
ZnT8	369 AA	Low to no expression in the brain	Cytoplasmic vesicle, secretory vesicle membrane, Cell membrane Chimienti et al. (2004), Chimienti et al. (2006)
ZnT9	568 AA	Cerebral cortex Hasna et al. (2019)	Mitochondrion membrane Kowalczyk et al. (2021), Rensvold et al. (2022)
			Nucleus Sim and Chow (1999)
			Endoplasmic reticulum Perez et al. (2017)
ZnT10	485 AA	Spinal cord Kaneko et al. (2015)	Cell membrane Bosomworth et al. (2012), Leyva-Illades et al. (2014), Zhao et al. (2016)
		Basal ganglia Kambe et al. (2014)	Golgi apparatus membrane Nishito et al. (2016)
			Recycling endosome membrane, early endosome membrane Patrushev et al. (2012)

**TABLE 2:** List of ZIP transporters, including number of amino acids, major locations of expression in the brain as well as sub-cellular location.

Protein	Amino acids	Major location of protein expression in the brain	Subcellular location
ZIP1	324 AA	Brain stem, cerebellum, forebrain, and hippocampus Qian et al. (2011b)	Cell membrane Gaither and Eide (2001), Milon et al. (2001), Milon et al. (2006)
		Microglia Higashi et al. (2011)	Endoplasmic reticulum membrane Milon et al. (2001)
ZIP2	309 AA	Not known	Not applicable
ZIP3	314 AA	Brain stem, cerebellum, forebrain, and hippocampus Qian et al. (2011b)	Cell membrane Kelleher and Lonnerdal (2005)
			Apical cell membrane Kelleher et al. (2009)
ZIP4	647 AA	Choroid plexus, microglia Belloni-Olivi et al. (2009)	Cell membrane Kim et al. (2004), Mao et al. (2007), Ahern et al. (2019)
			Recycling endosome membrane Kim et al. (2004)
			Apical cell membrane Dufner-Beattie et al. (2003), Weaver et al. (2007)
ZIP5	540 AA	Not known	Not applicable
ZIP6	755 AA	Choroid plexus Chowanadisai et al. (2008), Aquino (2014)	Cell membrane Chowanadisai et al. (2008)
		Neurons Chowanadisai et al. (2008)	Cell projection, lamellipodium membrane Taylor et al. (2003)
			Apical cell membrane Chowanadisai et al. (2005)
ZIP7	469 AA	Cerebral cortex Tian et al. (2016)	Endoplasmic reticulum membrane Taylor et al. (2004), Woodruff et al. (2018), Fauster et al. (2019)
			Golgi apparatus, cis-Golgi network membrane Huang et al. (2005)
ZIP8	460 AA	Spinal cord Li et al. (2016)	Cell membrane Begum et al. (2002); Lin et al. (2018)
		Cerebral cortex Talukder et al. (2021)	Lysosome membrane Begum et al. (2002), Aydemir et al. (2009)
		Hippocampal neurons Ji and Kosman (2015)	Apical cell membrane Steimle et al. (2019) Basolateral cell membrane Steimle et al. (2019)
ZIP9	307 AA	Cerebral cortex, cerebellum and hippocampus Willekens and Runnels (2022)	Golgi apparatus, trans-Golgi network membrane Matsuura et al. (2009)
		Glioblastoma Munnich et al. (2016)	Cell membrane Thomas et al. (2014)
			Cytoplasm, perinuclear region Thomas et al. (2014)
			Mitochondria Thomas et al. (2014)
			Nucleus Thomas et al. (2014)
ZIP10	831 AA	Cerebral cortex Talukder et al. (2021)	Cell membrane Taylor et al. (2016)
		Spinal cord Taylor et al. (2007)	Apical cell membrane Landry et al. (2019)
		Medulla Bin et al. (2017)	
ZIP11	342 AA	Not known	Not applicable
ZIP12	691 AA	Choroid plexus, medulla oblongata, and spinal cord Bin et al. (2017)	Membrane Zhao et al. (2015)
ZIP13	371 AA	Not known	Not applicable
ZIP14	492 AA	Spiral ganglion neurons Ding et al. (2014)	Cell membrane Taylor et al. (2005), Aydemir et al. (2016), Tuschl et al. (2016)
		Hippocampal neurons Ji and Kosman (2015)	Apical cell membrane Steimle et al. (2019)
			Basolateral cell membrane Scheiber et al. (2019), Steimle et al. (2019)
			Early endosome membrane Zhao et al. (2010), Aydemir et al. (2016)
			Late endosome membrane Aydemir et al. (2016)
			Lysosome membrane Zhao et al. (2010)
